# NBSP: an online centralized database management system for a newborn sickle cell program in India

**DOI:** 10.3389/fdgth.2023.1204550

**Published:** 2023-09-13

**Authors:** Apoorva Pandey, Sapan Borah, Bhavik Chaudhary, Shweta Rana, Harpreet Singh, Anita Nadkarni, Harpreet Kaur

**Affiliations:** ^1^ Division of Epidemiological and Communicable Diseases, ICMR Hqrs., Ramalingaswami Bhavan, Delhi, India; ^2^ Department of Haematogenetics, ICMR-National Institute of Immunohaematology, Mumbai, India; ^3^ Department of Biotechnology, National Institute of Pharmaceutical Education and Research (NIPER)-Ahmedabad, Gandhinagar, Gujarat, India; ^4^ Division of Biomedical Informatics, ICMR Hqrs., Ramalingaswami Bhavan, Delhi, India

**Keywords:** newborn screening, early diagnosis, sickle cell disease, data management, electronic health records, electronic data collection, hemoglobinopathies, India

## 1. Introduction

Sickle cell disease (SCD) is one of the most common monogenic disorders of hemoglobin, resulting from a point mutation (Glu > Val) at the 6th position of β-globin gene. Annually, over 300,000 babies are born with SCD worldwide, and these numbers are expected to increase from 305,800 in the year 2010 to 404,200 in 2050 (1). Most of these babies are born in Sub-Saharan Africa and India (1, 2). India has the highest prevalence of SCD in South Asia and more than 20 million sickle cell affected individuals reside in the country (3). The clinical complications associated with SCD involve severe hemolytic anemia, splenic dysfunction, painful crisis and bacterial infections. Early detection of SCD is important as it has potential in the management of disease severity and reduction in mortality & morbidity (3).

Web-based systems for clinical data collection are rapidly expanding in healthcare research. Advantages include real-time data entry, accuracy, accessibility and integration with electronic health records (EHR). They have been applied in fields like neonatology, cardiovascular electrophysiology and epidemiology. Continued research and innovation in this field will optimize healthcare research and patient outcomes. Despite the availability of several Electronic Data Collection (EDC) platforms as self-platforms such as Oracle Clinical (4), ClinTrial (5), eClinical (6), REDCap (7), PROMIS (8), etc. or networks (9), their use is limited due to specific requirements of the researchers (9, 10). Shanbehzadeh et al. (11) described the development of EDC using REDCap in the field of cardiology, which significantly improved the efficiency of clinical documentation. The Ligurian Infectious Diseases Network (LIDN) (12) provides a good example of the scope of expansion of small and disease specific EHRs. Initially developed for a regional multicenter clinical trial for a HIV drug, the LIDN was gradually integrated into laboratory information systems for direct uploading of clinical tests to reduce manual data entry and errors. The LIDN architecture was further expanded for use in other diseases and automatic data collection from different hospitals. In India, there are two major programs where sickle cell screening data is captured electronically. First, under a state-wide sickle cell screening program in the Indian state of Chhattisgarh, an Electronic Medical Record (EMR) system was developed and implemented in nine districts of this state, which is an elaborate stand-alone windows-based software developed using VB.net. Between January 2014 and June 2015, the system captured data of 1,305,357 individuals who were screened for SCD (13).

Second, the National Sickle Cell Disease Control Programme initiated under the National Health Mission (NHM), Government of India, captures the demographic and testing details of individuals screened for SCD. This program has been recently launched in 17 states with high prevalence of SCD across the country and captures data through a mobile app. According to the dashboard, the program has screened 95,078 individuals as of 1st March 2023 (14).

As far as National prevalence of SCD is concerned, there is a lack of pan-India studies on the prevalence of SCD. However, scattered data is available and the state-wide sickle cell prevalence from published studies is shown in (Table 1) which also reflects the state-wide prevalence emerging from our program centers, the 7 centers through this study, in parallel. We have, however, not included the unpublished literature. Though efforts are being made to capture SCD screening data in some states of the country, there are limitations with both the above systems. Both the systems lack a provision for patient follow-up and immunization data. The source code of neither of these systems is available in public domain for implementation by any interested stakeholder (organization or non-governmental organization).

**Table 1:** Prevalence of sickle cell disease in Indian states.

Prevalence data from other studies [Numbers screened (Prevalence in %)]		Reference	Prevalence data from this study [Numbers screened (Prevalence in %)] (May 2019–February 2023)
Gujarat	3, 17,539 (0.31%)	(15)	SEWA Rural—8,117 (1.53%)
	5,467 (0.60%)	(16)	
	8,411 (1.45%)	(17)	
Madhya Pradesh	505 (1.18%)	(17)	NIRTH—9,721 (0.69%)
Odisha	761 (1.7%)	(18)	RMRC—6,036 (1.57%)
Maharashtra	19,833,217 (0.08%)	(19)	NIIH and NIRRCH—20,716 (0.48%)
Rajasthan	36,752 (0.17%)	(20)	NIIR-NCD—6,385 (0.54%)
Tamil Nadu	–	–	NAWA—2,170 (0.59%)
Chhattisgarh	38,472 (23%)	(21)	–

In this paper, we describe a web-based system developed by ICMR Hqrs., New Delhi, using open-source technologies for capturing the newborn sickle cell screening and follow-up data, which was initiated well before the above-mentioned NHM's screening program. This is ongoing since May 2019 at seven centres in six states of India and is aimed at the early detection of SCD and also providing early comprehensive care to SCD babies of India, particularly from tribal populations. The source code for this program is available in the git repository for implementation and to the best of our knowledge; this is the first system in India which is available as a downloadable version for use by the interested stakeholder.

Recognizing the prevalence of SCD and the importance of screening, the Government of India in its annual budget 2023–2024, has proposed a mission to eliminate SCD in India by 2047. In this regard, we believe that our pilot study could serve as a model for a pan-India screening program as it demonstrates the usefulness of an online data portal in sickle cell disease management.

## 2. Construction and content

### 2.1. System architecture and technology

The Newborn Sickle Cell Portal (NBSP) is a web-based system/tool developed using open-source technologies and hosted on the Docker Container Version 20.10.12, Ubuntu 20.04 Operating System using a Gunicorn, Nginx Web Server. This system is developed using Python (Django) version 4.0 database as a back-end software tool and CSS, Javascript, Bootstrap etc., as front-end tools. The visualization is developed using HTML and graphics using chart JS.

The tool has a modular architecture with features encompassing: (i) centralized data entry; (ii) role-based access to data and dashboard; (iii) data import/export through MS-Excel and (iv) reminder to the centres for patients who miss follow-ups.

**Figure 1:**
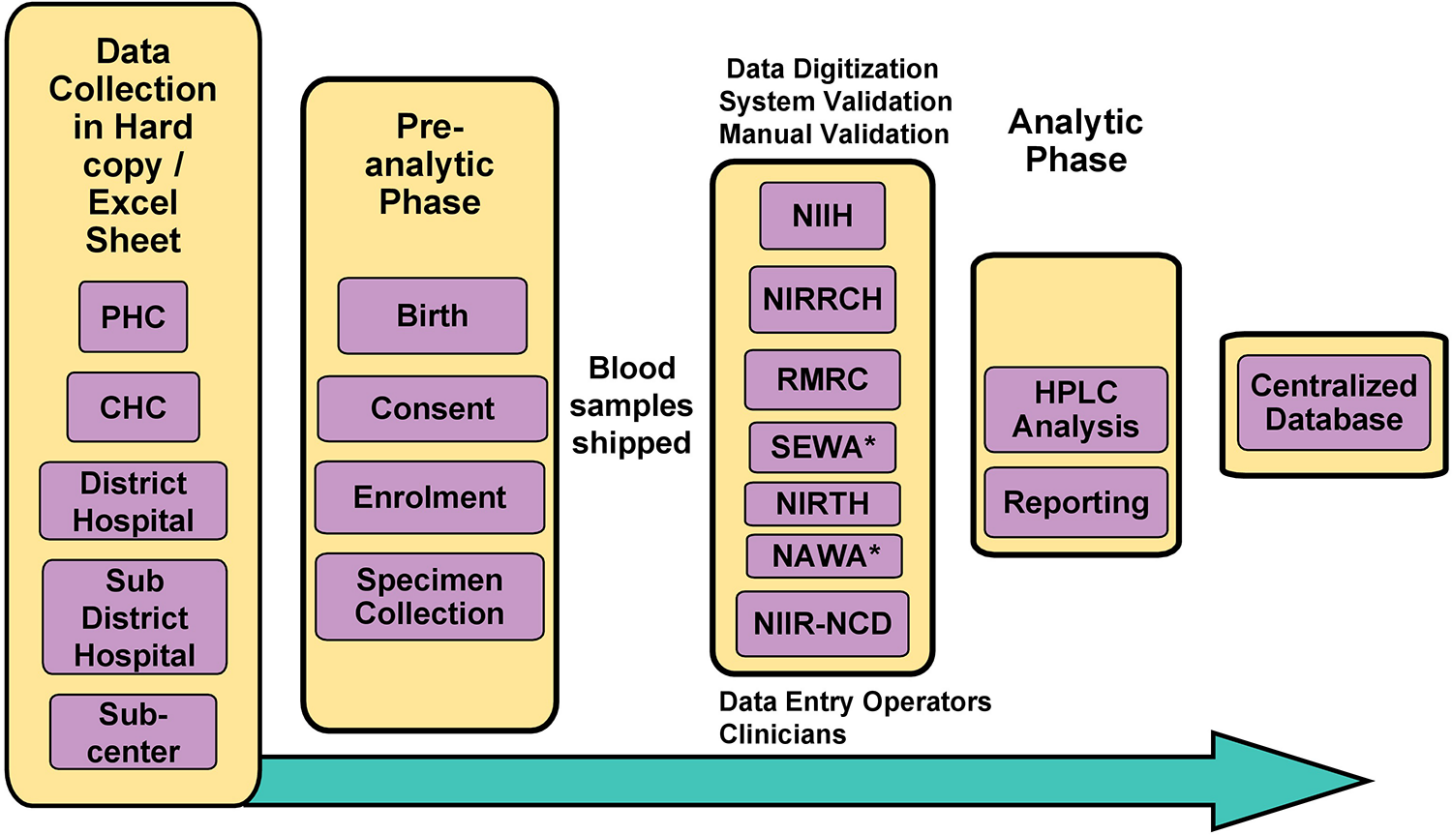
Flowchart outlining the system’s architecture of NBSP. *Non-ICMR Institutes (Non-governmental Organization); CHC, Community Health Centre; PHC, Primary Health Centre; HPLC, High-performance Liquid Chromatography.

A comprehensive flow-chart describing the system's architecture is shown in Figure 1. The developed system is being used by the ICMR newborn sickle cell screening program and is available at https://nbsp.icmr.org.in. As this is an ongoing study and the data has not been analysed and published, we have not made the portal available to the public yet.

### 2.2. Database structure

The database primarily comprises of four tables: (i) Registration table, which captures information related to the demography and sickle status of the newborn screened in the form of HPLC reports; (ii) Repeat Screening table stores information related to confirmation of the sickle status; (iii) Follow-up table stores the clinical information of the sickle cell newborn babies and tracks appointments, progress etc., and (iv) Immunization table that stores immunization history of the newborns who are being followed up, thus helping ensure timely vaccinations and monitoring the immunization coverage.

Additionally, there are a few other tables for access logs and audit trails. These tables track and record information about database access and changes made to maintain data security and integrity.

By organizing the data into these tables, the database facilitates efficient storage, retrieval, and management of information related to registration, repeat screenings, follow-ups, and immunization. It promotes better coordination and analysis of data, enabling healthcare professionals to make informed decisions.

### 2.3. Data source and collection

Due to challenges involving connectivity in remote tribal locations of this study, the data is captured in printed data collection forms (DCF) at the sites of sample collection by the Medical Social Workers (MSWs). The DCFs were specifically designed for the current newborn screening program in order to maintain uniformity in data collection across all the centers. The same has been digitized in the system. The data from DCF is entered into the system at the regional centres by Data Entry Operators (DEOs). This collection module is flexible and the data can be entered through an online platform using a built-in integrated form or uploaded using MS-Excel. In addition to capturing demographic details (Supplementary Figure S1) of the screened individuals in this system, an online entry form has three parts viz. baseline information, follow-up, and immunization information of the enrolled individuals in the program. All the system attributes configured by the Super Administrator (ICMR Hqrs., New Delhi) are visible in this form and the data entry process is compact & user-friendly.

The system has a basic data analysis module, which provides varied information related to role-based visualization of state/district-wise prevalence, mean age of the gestation period, number of total enrolments, follow-up data, immunization records etc.

### 2.4. Data storage and security

The application's data is securely stored in a centralized storage area network (SAN) system. Till date, the system houses over 60,000 records and receives a significant number of submissions daily. To ensure the utmost data security, the Institution employs a robust security protocol. This includes a customized firewall that acts as a safeguard against unauthorized access. Additionally, the storage is protected by restricted access measures, allowing only authorized personnel to handle and retrieve the data. These stringent security measures guarantee the confidentiality and integrity of the stored information.

### 2.5. NBSP database

**Figure 2:**
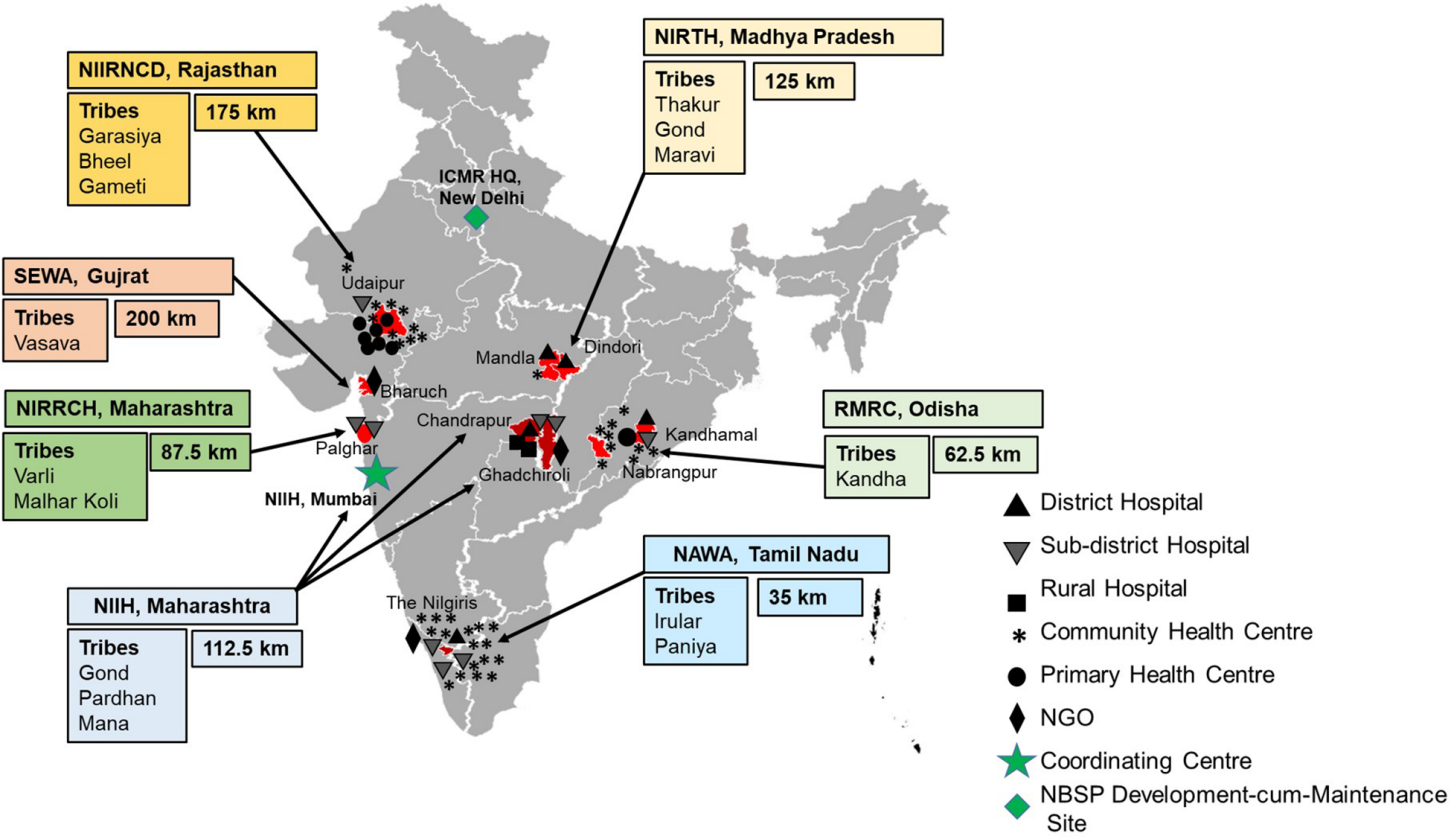
Map depicting the locations of newborn screening centres across India. Boxes indicate the name of the participating screening centre, name of the major ethnic tribes in the area and average distance travelled by SCD individuals to the centre. Black and Grey symbols (Triangle, Inverted Triangle, Square, Asterisk, Solid Circle, and Rhombus) indicate the number and kind of health facility of newborn blood collection centre. Green Rhombus indicates the location of the online portal Development cum Maintenance Site, ICMR, New Delhi. The Green Star shows the location of the Coordinating Centre NIIH, Mumbai. The map is not to scale and is for representational purpose only.

**Figure 3:**
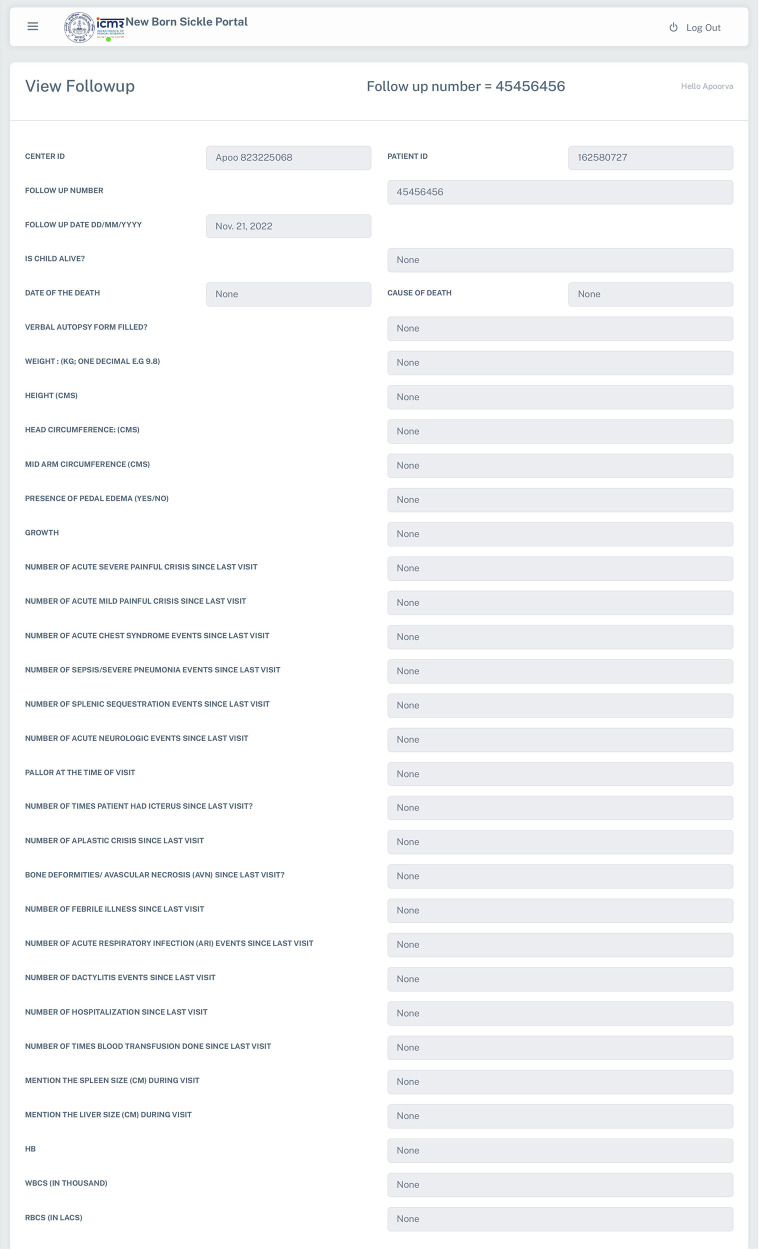
A snapshot of the follow up form in the NBSP.

The web-based system for collection, management and analysis of data collected from the ICMR's Newborn screening Program was launched in the year 2019 at seven sites namely ICMR-National Institute of Immunohaematology (NIIH, Mumbai, Maharashtra); ICMR-National Institute for Research in Reproductive and Child Health (NIRRCH, Dahanu in Palghar, Maharashtra); ICMR-Regional Medical Research Centre (RMRC, Bhubaneswar, Odisha); Society for Education, Welfare and Action–Rural, (SEWA, Bharuch, Gujarat); ICMR-National Institute of Research in Tribal Health (NIRTH, Jabalpur, Madhya Pradesh); ICMR-National Institute for Implementation Research on Non Communicable Diseases (NIIR-NCD, Jodhpur, Rajasthan); Nilgiris Adivasi Welfare Association (NAWA, Kotagiri, Tamil Nadu), apart from the ICMR Hqrs., in Delhi, which is the Development cum Maintenance Site and acting as the Super Administrator for the Portal, as represented in Figure 2. Under the program, demographic details include data about neonates along with parent's details, health status, gestational age at birth, birth weight, etc. From May 2019 to February 2023, the Portal has enrolled and collected data from 53,145 neonates, of which 27,320 (51.40%) are males and 25,825 (48.59%) are females. The Portal has captured clinical data of 1,902 follow-ups & 296 immunization data. There has been a steady increase in the number of entries in every year, viz., 2023 (3,396 entries until February 28th), 2022 (22,567 entries), 2021 (13,677), 2020 (9,209), and 2019 (4,296). A cohort of 437 babies has been identified under the screening program and is receiving regular clinical interventions. The follow up form records the details of anthropometry (weight, height, head and mid-arm circumference and growth), complete blood count (CBC), clinical complications, hospitalization, HPLC analysis, and clinical interventions (antibiotics, vaccination, iron supplementation, and hydroxyurea) (Figure 3).

### 2.6. Unique identification number

The identification system of the NBSP gives a unique centre ID and patient ID to each newborn screened, both of which are system generated. The first four alphabets in the centre ID are representative of each study site, which is followed by a unique 8-digit numerical sequence. Likewise, the patient ID has a unique 9-digit numerical sequence. These unique numerical sequences make sure that there is no overlap of the screened individuals with those of the sickle cell diagnosed babies or with traits that are thus recognized eventually for follow-up.

### 2.7. Data validation module

Once the child is diagnosed with SCD, lack of standardized follow-up practices in a remote and resource-poor setting like India may lead to variability in the data. To keep the data uniform across all the centres, and minimise human errors in data entry, each clinical parameter is provided with a range.

## 3. Utility and discussion

In a low income and high burden setting like India, SCD is a major public health challenge where about 20% of children with SCD die before reaching the age of 2 (3, 22). In this context, the Newborn screening aids in the early identification of SCD. However, for a screening program to be successful, it is essential that the identified SCD newborns are followed up systematically with regular clinical interventions and that the data collected is uniform & curated for detailed analysis. However, undertaking such a screening program in a resource-limited setting like India has challenges in the form of lack of medical, technical and logistical support. Under the present ICMR initiated newborn screening sickle cell program, which is ongoing since May 2019 in six states of India, we built a web-based data management system namely NBSP using open-source technologies to capture the newborn sickle cell screening, follow up, immunization, and demographic data.

The NBSP system has a modular architecture with minimal hardware requirements, so it can be easily integrated into the existing laboratories in the country. The system has several advantages over other current and previous sickle cell screening data management systems in India. First, it records the data in a centralized manner thus easing up data management and analysis. Second, it holistically captures the demographic & screening details, follow-up and immunization data. Third, as the system is developed using open-source technologies, it is cost-effective and easily accessible to interested stakeholders. Finally, the availability of the source code of the system on git repository facilitates field ease of implementation by stakeholders. In addition to the SCD status of the newborns, the NBSP also has a provision to collect data on other hemoglobinopathies. To protect the privacy of the NBSP data, access to patient information in the project is granted, based on roles and hierarchy. Different security roles are assigned to users to control their level of access and ensure data protection. By implementing a role-based hierarchical access control system, NBSP ensures that users have appropriate access privileges based on their roles and position within the organization.

While the NBSP system encompasses several advantages, due to challenges involving connectivity in remote tribal locations, the system suffers from minor limitations as it captures data in printed DCF at the sites of sample collection. Although the data can be entered online using a built-in integrated platform or uploaded using Excel, this introduces an additional step in the data capture process. However, the system is still in the development phase, and these features leave a scope of improvement in future.

The initial implementation of NBSP at the remote study sites was challenging and multiple training sessions were imparted to the DEOs, MSWs and other staff. Based on the feedback from the end-users, several improvements were made to the NBSP to make it a more compact, user-friendly, and implementable platform. It has facilitated data entry and workload sharing by the sites locally, while simultaneously allowing the Development cum Maintenance Site (Super Administrator) in Delhi to identify and eliminate specific data inconsistencies and clarify ambiguities. The data generated through this system is readily providing information on the newborn screening program, which would not only help in better understanding of both the disease characteristics as well as its progression through secondary data research, but subsequently, also pave way for future research and policy-making decisions by the relevant stakeholders.

## 4. Conclusions

NBSP is an innovative and cost-effective tool for sickle cell screening and follow-up. This portal has been nascently developed as a part of an ongoing study of ICMR on newborn sickle cell screening and is important in future, considering the burden of sickle cell in the country, to be upscaled with added new features and further integration for use under the Govt of India's Mission launched to eliminate sickle cell by 2047.

Fast Healthcare Interoperability Resources (FHIR) is a standard interface, developed by Health Level Seven International (HL7) to enable electronic exchange of healthcare information. The purpose of this interface is to enhance interoperability and facilitate standardized communication with other systems in the healthcare industry and is designed to streamline the exchange of healthcare data, contributing to faster healthcare delivery and improved comprehensive care. Future versions of the tool will include the implementation of HL7 FHIR for smoother integration with other systems. Further, as standard terminology is important for deriving data from several sources, we plan to implement the same in the future release.

## Data Availability

The original contributions presented in the study are included in the article/**Supplementary Material**, further inquiries can be directed to the corresponding author.

## Supplementary material

SUPPLEMENTARY FIGURE S1

A snapshot of the Enrolment form in the NBSP.
