# Supplementary figures and images for: NBSP: an online centralized database management system for a newborn sickle cell program in India
Source: Front Digit Health. 2023 Sep 13;5:1204550. doi: 10.3389/fdgth.2023.1204550 (PMC10534972; doi:10.3389/fdgth.2023.1204550)

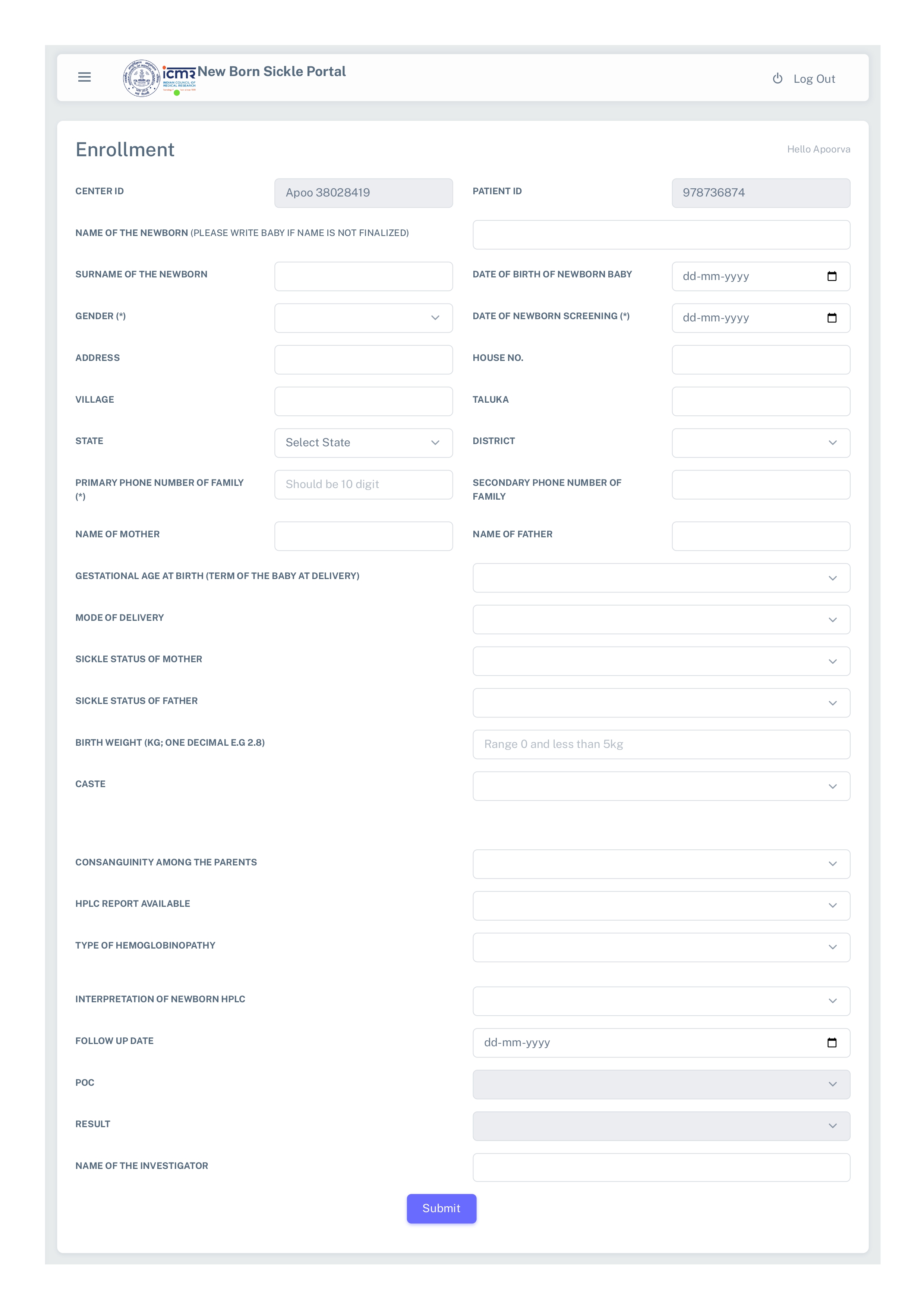

Supplement: Supplementary file 1 [file Image1.jpeg]
